# The transcriptome of the rat subfornical organ is altered in response to early postnatal overnutrition

**DOI:** 10.1016/j.ibror.2018.06.001

**Published:** 2018-06-26

**Authors:** Colleen S. Peterson, Shuo Huang, Samantha A. Lee, A.V. Ferguson, W. Mark Fry

**Affiliations:** a Department of Biological Sciences, University of Manitoba, Winnipeg, MB, R3T 2N2, Canada; b Centre for Neuroscience, Queen’s University, Kingston, Ontario, K7L 3N6, Canada

**Keywords:** Subfornical organ, RNA sequencing, Transcriptome, Microarray, Postnatal overnutrition

## Abstract

Early postnatal overnutrition in humans is associated with long-term negative outcomes including obesity, increased risk of type-II diabetes, and cardiovascular disease. Hypothalamic neurons from rodents exposed to early postnatal overnutrition show altered expression of satiety signals and receptors, and exhibit altered responses to many satiety signals, suggesting a hypothalamic link between early overnutrition and development of these sequelae. Importantly, several hypothalamic nuclei receive information regarding circulating hormones (such as insulin, leptin and ghrelin) from the subfornical organ (SFO), a forebrain sensory circumventricular organ which lacks a blood brain barrier. Previous transcriptomic studies indicate that challenges to energy balance and hydration status stimulate changes in gene expression within the SFO, including genes encoding ion channels and receptors. In order to determine if early postnatal overnutrition also causes changes in SFO gene expression which may be associated with homeostatic dysregulation, we performed whole transcriptome sequencing on SFO tissue from rats raised in small (4 pups), or control (large, 12 pups) litters. Illumina RNA sequencing was performed on SFO tissue from rats raised from small and large litters, and read sequences were aligned to the Rat Rnor_6.0 genome. Control data were further compared to previously published microarray data set for validation. We found statistically significant (p < 0.05) changes in expression of 12 transcripts, three of which have likely roles in neuronal excitability, neurite outgrowth and differentiation, and food intake (*Manf, Slc24a4, Cracr2b*). Additionally, gene ontology analysis identified a trend among significantly altered transcripts in roles for oxidative stress response. We conclude that the SFO transcriptome is subtly altered by early postnatal overnutrition, and recommend further investigation of the effect of early postnatal overnutrition on SFO physiology and morphology.

## 1 Introduction

In humans, early postnatal overnutrition results in accelerated growth and weight gain, is predictive of childhood (Gittner et al., 2013) and adult obesity (Rzehak et al., 2017), and is correlated with increased risk of type II diabetes (Eriksson et al., 2003) and heart disease (Camhi and Katzmarzyk, 2010). Similar effects are seen in rodents raised in small litters as a model of early postnatal overnutrition: rats from small litters gain more weight relative to controls (Wiedmer et al., 2002), a trend which persists into adulthood (Habbout et al., 2013). Moreover, rodents show increased visceral white adipose tissue (Cordeiro et al., 2009), hypertension (Plagemann et al., 1992), hyperinsulinemia (Plagemann et al., 1992), and hyperleptinemia (Plagemann et al., 1999a) following early postnatal overnutrition. When fed a high fat diet, small litter rats display persistent hyperphagia and increased caloric intake (Glavas et al., 2010), suggesting early overnutrition primes rats for later weight gain. Numerous studies indicate that altered neurophysiological development plays a key role (Glavas et al., 2010; Habbout et al., 2013; Rodrigues et al., 2009). Increased densities of galanin and neuropeptide Y (NPY) neurons in the arcuate nucleus of the hypothalamus (ARC) are found in small litter rats compared to control rats, despite lower overall density of neurons (Plagemann et al., 1999b). Galanin neuron density is similarly increased in the paraventricular nucleus of the hypothalamus (PVN; Plagemann et al., 1999a). Neurons may additionally show altered responses to hormones following postnatal overnutrition; for example leptin and insulin resistance is seen in hypothalamic neurons (Glavas et al., 2010) in the face of increased central levels of both peptides (Glavas et al., 2010; Plagemann et al., 1999b). Altered responses to the neuropeptides cocaine and amphetamine regulated transcript (CART), NPY, and corticotropin-releasing hormones are also observed in the PVN, ARC, and ventromedial nucleus of the hypothalamus (VMN) (Davidowa et al., 2003; Davidowa and Plagemann, 2004)

The subfornical organ (SFO) is a small forebrain sensory circumventricular organ found on the anteroventral aspect of the third ventricle, between the columns of the fornix and attached to the hippocampal commissure. Sensory circumventricular organs are specialised structures uniquely lacking a blood-brain barrier (Gross and Weindl, 1987). They express a wide variety of receptors for circulating hormones and signaling molecules, which when activated modulate electrical activity of SFO neurons to allow transduction of information from the periphery to the brain (Fry and Ferguson, 2007). The SFO receives synaptic inputs from homeostatic regulatory regions including the nucleus of the solitary tract (NTS) (Tanaka et al., 1997), the lateral hypothalamus (LH) (Lind et al., 1984), and the median preoptic nucleus (MPN) (Lind et al., 1984). The SFO also sends axonal projections to various other centres including the PVN (Miselis, 1982), ARC (Gruber et al., 1987; Whyment et al., 2004), the LH (Burton et al., 1976; Miselis, 1982), the MPN (Lind et al., 1984, 1982), the supraoptic nucleus (SON) (Lind et al., 1982; Sawchenko and Swanson, 1983), and the vascular organ of the lamina terminalis (OVLT) (Lind et al., 1982). Critical roles for the SFO have been established in osmoregulation (Felix, 1976), cardiovascular output (Mangiapane and Simpson, 1983), and energy homeostasis (Smith et al., 2010). Stimulation of the SFO results in feeding in satiated rats (Smith et al., 2010), and microarray data shows strong expression of receptors for hormones involved in energy balance including adiponectin, leptin, amylin, and ghrelin, the activity of which is confirmed in SFO neurons (Hindmarch et al., 2008, and references therein).

That the SFO plays a key role in integration of signals related to energy balance and cardiovascular regulation is highlighted by the observation that acute fasting or water restriction dramatically modulates its transcriptome, causing significant alteration of hundreds of transcripts (Hindmarch et al., 2008). Interestingly, research performed on the impact of postnatal overnutrition on the brain has focused on other nuclei such as the ARC, (Davidowa and Plagemann, 2000; Juan De Solis et al., 2016), VMN (Davidowa et al., 2002), and the PVN (Davidowa and Plagemann, 2004; Plagemann et al., 1999a), but not the SFO which is a key centre communicating information from the periphery to the hypothalamus. In order to evaluate possible roles of SFO in the long term effects of early postnatal overnutrition, we compared the transcriptomes of the SFO of six-week-old male rats raised in small litters (4 pups) to those raised in larger (control) litters (12 pups) using RNA sequencing. Here we report statistically significant (false discovery rate corrected, p < 0.05) 1.16–1.7 fold changes (in either direction) in 12 SFO transcripts involved in neuronal excitability, neuron differentiation and outgrowth, food intake, and/or response to oxidative stress, from rats raised in small litters compared to those raised in large litters.

## 2 Methods

### 2.1 Animals and diets

All procedures were approved by the University of Manitoba Animal Care Committee in accordance with the Canadian Council for Animal Care. Two groups of 8 timed-pregnant dams, all birthing on the same day were used for these studies. For each group of 8 dams on postnatal day one (PD 1), all of the pups were mixed and randomly assigned to a dam: 2 dams received 12 pups to form a large litter, whereas 6 dams received 4 pups to form a small litter. The litters were composed of equal numbers of male and female pups. This procedure was repeated a second time with a separate set of 8 dams and their pups, for a total of 16 dams; 4 large litters and 12 small litters. Weights between control and small litter rats (n = 48, male and female each) on PD 3 were 12.3 ± 0.3 g and 12.9 g ± 0.3 g respectively were not significantly different (p > 0.05, *t*-test).

After weaning, rats were kept in a light-controlled facility (12 h light: 12 h dark), and given ad libitum access to standard rat chow. Pups were weaned at PD 21 into cages of two males or two females.

Male rats were sacrificed on PD 42–47, between the hours of 9 a.m. and 12 p.m. (2:45–5:45 following lights on). Briefly, rats were decapitated, the brain removed and placed in ice-cold oxygenated artificial cerebrospinal fluid (composition, in mM: 126 NaCl, 2.5 KCl, 1.25 NaH_2_PO_4_, 2 MgCl_2_, 10 d-glucose, 26 NaHCO_3_, 0.5 CaCl_2_) for 2 min. A 3 mm block of tissue was cut at the level of the hypothalamus (approximately 9–6 mm interaural in the coordinates of the Paxinos and Watson rat brain atlas (Paxinos and Watson, 2007)), and a vibratome was used to prepare a brain slice containing the SFO, which was dissected out, using caution not to harvest choroid plexus. Brain tissue was stored in RNAlater at −20 °C until time of RNA extraction. Only SFO tissue from male rats was used for RNA sequencing. To reduce biological variability, SFO tissue was pooled (Colombari et al., 2011; Hindmarch et al., 2008, 2006). Briefly, 6 males from each large litter were pooled in to a single RNA sample for sequencing, providing 4 large litter samples of 6 SFO each. For small litters, the 2 males from the 3 litters (for a total of 6 SFO) were pooled, resulting in 4 small litter samples of 6 SFO each.

### 2.2 RNA extraction and sequencing

RNA extraction and sequencing were performed at the ABM laboratory in Vancouver. RNA extraction was performed using the PureLink RNA Mini Kit (Thermo Fisher Scientific), and analysed using a Bioanalyzer (Agilent). RNA integrity number values (RIN) were assessed using the RNA 6000 pico kit on an Agilent 2100 Bioanalyzer; all RIN values were between 7.7 and 8.6. Two micrograms of sample were prepared for library construction using the TruSeq Standard mRNA Library Prep Kit (Illumina). Briefly, the RNA was subject to polyA enrichment, fragmentation, and cDNA synthesis. Indexed adaptors were ligated to the cDNA library, and then libraries were pooled in equal molar ratio. Quality of library was assessed using an Agilent Bioanalyzer prior to sequencing; library sizes ranged from 401 to 427. Sequencing was performed on the NextSeq 500 platform with 2 × 75 bp end sequencing. For each sample, sequencing produced between 42.8 and 54.7 million reads: reads were aligned to Rat Rnor_6.0 reference genome and annotation (GCF_000001895.5) using Hisat2 with the parameters (--rf --mm --dta); mapping rates were between 86.76% and 88.52%. Stringtie was used to estimate FPKM for each sample and differential expression between small and control litter rats was performed using DESeq2. Differentially expressed genes were identified according to log_2_(fold change) > 0.5 or < -0.5 with adjusted p-values < 0.05 (Wald test with Benjamini-Hochberg multiple test correction, as previously described (Love et al., 2014)). Functional annotation with gene ontology and pathway annotation was performed using KOBAS (Xie et al., 2011).

### 2.3 Validation of RNA sequencing data

Relative expression of transcripts identified by RNAseq in control rats were compared against those from the control group of an existing microarray dataset (Hindmarch et al., 2008).

The rats used in the experiments performed by Hindmarch et al. were slightly older (10–12 week) male Sprague Dawley rats from Harlan Sera-lab, Loughborough, UK; litter size is unknown. There were also minor differences in sample collection: dissections occurred at slightly different times in the morning (9 A.M.–12 P.M. in our study vs 11 A.M.–1 P.M.), rat brains used in this study were cooled in aCSF before SFO dissection whereas those from Hindmarch’s study were immediately placed in a cooled brain matrix post-removal. In both cases, choroid plexus was removed and the SFO was carefully dissected out under microscope from a larger slice, however Hindmarch et al. used a single 1 mm section, whereas we have used a larger 3 mm section, which was then cut on a vibratome to reveal the SFO. In both studies, samples were stored in RNAlater at −20 °C.

Ensembl Gene ID was used as the annotation feature for matching unique genes from each experiment. To allow for fair comparison between microarray and RNAseq data, only those transcripts which could be mapped back to the microarray were included. Of the 31,100 probe sets present on the GeneChip Rat Genome 230 2.0 Array, 12,779 were not annotated with Ensembl gene IDs and were excluded from analysis, whereas 10,910 probes were annotated both with Ensembl gene IDs (of which 8914 were unique Ensembl IDs) and marked as present on the microarray. In the case of RNA-sequencing data, 15,628 transcripts with associated Ensembl gene IDs were considered present (FPKM > 0.5), and for comparison of the two datasets, only the transcripts with Affy IDs and associated Ensembl IDs which were detected and called as present by both methods were included.

## 3 Results

### 3.1 Effect on body weight of early overnutrition

To confirm that rearing as small litters induced larger body weight, males were weighed at six weeks of age. Consistent with previously published observations (Habbout et al., 2013, 2012), male rats raised in small litters showed significantly increased body weight relative to control rats, with weights of 418.5 ± 4.5 g and 382.9 ± 6.9 g for small and control litters respectively (p = 7.6 × 10^−5^, *t*-test).

### 3.2 Differential expression analysis

Of the 32,884 transcripts identified for the Rat Rnor 6.0 genome assembly, 15,630 were identified as present in the SFO. To determine the effect of postnatal overnutrition on the transcriptome of the SFO, differential expression analysis was performed using DESeq2. Statistically significant changes in relative expression were detected for 12 transcripts (p < 0.05, with multiple test correction). Specifically, 4 transcripts were upregulated and 8 were downregulated in response to postnatal overnutrition. Gene ontology terms were found in oxidative stress response, and regulation of protein folding/activity were detected by KOBAS gene ontology analysis (Table A1).

### 3.3 Comparison to microarray data

In order to validate the present RNAseq experiment, sequencing data from control litters was compared to control data from previously published microarray data from the SFO (Hindmarch et al., 2008). Of the 18,321 Affymetrix probe IDs which could be mapped to Ensembl IDs, 10,910 were detected by the microarray; these mapped to 9115 unique Ensembl IDs. Of those, only 174 transcripts were detected by the microarray, but were not detected by RNA sequencing. Conversely, 3586 transcripts were detected exclusively by RNA sequencing (Fig. 2), suggesting higher sensitivity. Relative expression (FPKM and relative intensity) of transcripts present in both data sets were compared (Fig. 3), and were demonstrated to have good concordance (Pearson’s *r* = 0.65455).

## 4 Discussion

**Fig. 1:**
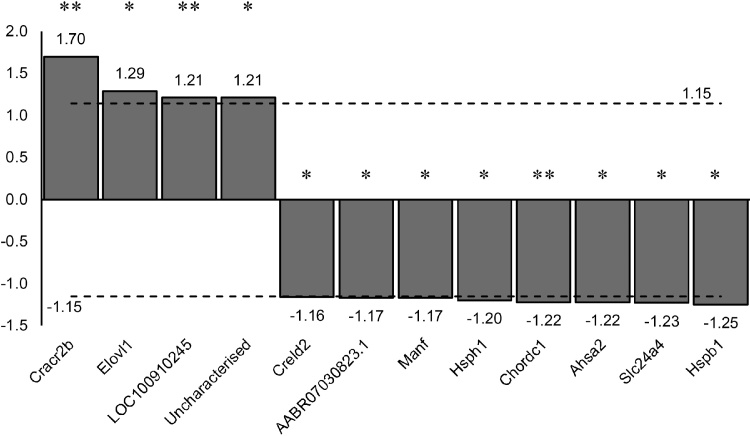
Fold regulation of transcripts significantly changed (p < 0.05; * indicates p < 0.05; ** p < 0.01) by postnatal overnutrition in rat subfornical organ. *Cracr2b* = EF-hand calcium-binding domain-containing protein 4A; *Elovl1* = Elongation of very long chain fatty acids protein 1; *LOC100910245* = Ribose-phosphate pyrophosphokinase 2-like; *Creld2* = Cysteine-rich with EGF-like domain protein 2; *AABR07030823.1* is a pseudogene; *Manf* = Mesencephalic astrocyte-derived neurotrophic factor; *Hsph1* = Heat shock protein 105 kDa; *Chordc1* = Cysteine and histidine-rich domain-containing protein 1; *Ahsa2* = AHA1, activator of heat shock protein ATPase 2 (p = 0.01485); *Slc24a4* = Solute carrier family 24 member 4; *Hspb1* = Heat shock 27 kDa protein 1. Dotted lines indicate fold-change cut-offs, greater than 1.15 fold up- or downregulation.

The objective of this study was to determine if there are changes in SFO gene expression associated with early overnutrition in rat pups that may be related to the development of sequelae such obesity, type II diabetes (Eriksson et al., 2003) and heart disease (Camhi and Katzmarzyk, 2010) observed in humans and rodents (Habbout et al., 2013; Plagemann et al., 1992). We observed statistically significantly differential expression in 12 transcripts (Fig. 1); of these one was identified as a pseuodogene (*AABR07030823.1*) and one is currently uncharacterised. Of particular interest are three transcripts, mesencephalic astrocyte-derived neurotrophic factor (*Manf*), EF-hand calcium-binding domain-containing protein 4A (*Cracr2b*), and solute carrier family 24 member 4 (*Slc24a4*), as they may alter neuronal excitability or development. Ultimately we found only subtle changes in few transcripts, leading us to conclude that the SFO does not show the dramatic changes following early postnatal overnutrition compared to changes elicited by acute food or water restriction (Hindmarch et al., 2008).

**Fig. 2:**
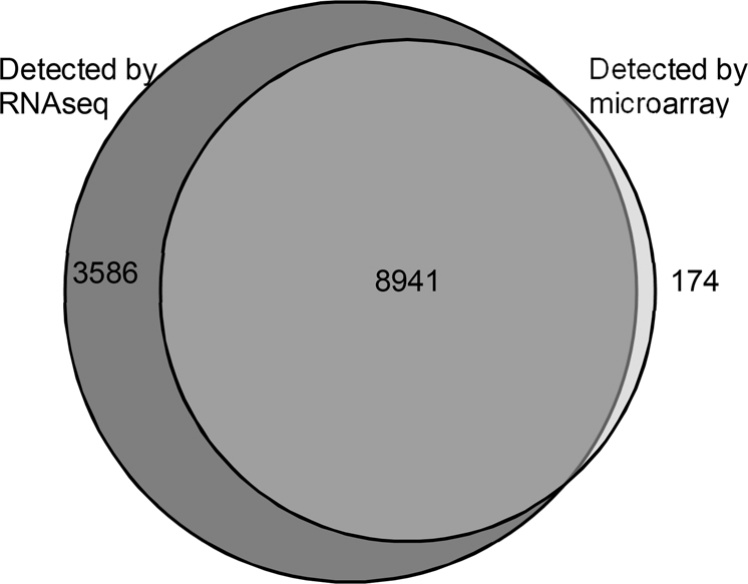
Overlap between previously published microarray data and RNA sequencing transcriptomes. Only transcripts which could be mapped between the RNAseq dataset and microarray dataset via Ensembl IDs were included. 8941 unique transcripts (unique Ensembl IDs) were detected by both the microarray and RNAseq. 3586 and 174 transcripts were exclusively detected by RNAseq and microarray respectively.

**Fig. 3:**
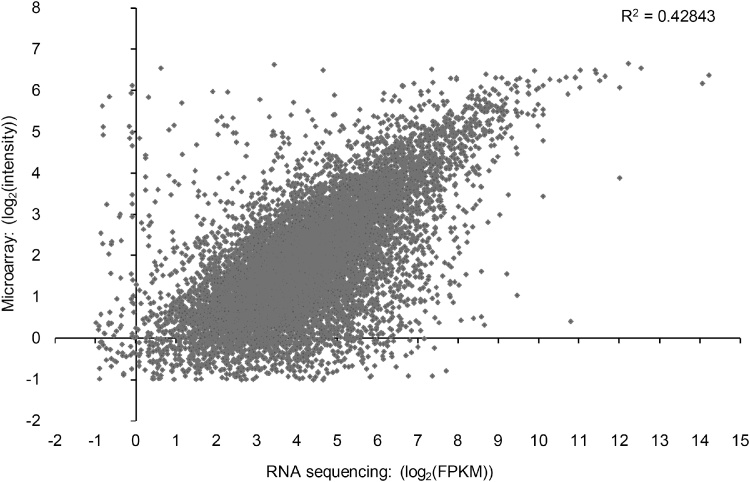
Comparison of rat subfornical organ relative expression (FPKM) values to publicly available rat subfornical organ Affymetrix microarray relative expression (intensity) (Hindmarch et al., 2008). Relative expression levels were determined based on log2 transformed average expression values in all control samples in each experiment. 8941 unique Ensembl gene ID annotated transcripts detected by both methods are included; of these 1969 Affy IDs map to the same Ensembl identified as at least one other Affy ID, such that 10,910 points are included. Good concordance exists between the two methods despite differing experimental conditions, as demonstrated by the Pearson correlation coefficient (*r =* 0.65455).

*Manf* is a neurotrophic factor whose roles in the CNS have yet to be fully elucidated. Increasing evidence suggests Manf participates in neurite outgrowth and neuronal differentiation (Tseng et al., 2017) as well as protection against cell damage in Parkinson’s disease (Voutilainen et al., 2009), cerebral ischemia (Airavaara et al., 2009), and endoplasmic reticulum stress (Apostolou et al., 2008; Hellman et al., 2011). Recent data suggests that Manf, acting at the level of the PVN, plays a role in regulation of energy balance. Specifically Manf protein and transcript levels rise in response to fasting and fall following feeding; Manf overexpression or direct injection to the hypothalamus causes hyperphagia and increased body weight, whereas Manf knockdown results in hypophagia and decreased weight gain in 3 month old mice (Yang et al., 2017). Whether Manf also acts to negatively regulate energy balance via the SFO is unknown. Additionally, given its roles in neurite outgrowth and neuronal differentiation in the cortex (Tseng et al., 2017), there is also potential for altered SFO neuron development resulting from decreased *Manf* expression.

*Slc24a4* is a member of a family of potassium-dependent Na+/Ca++ exchangers, exchanging one Ca++ ion and one K+ ion for four Na+ ions (Schnetkamp, 1995). Altered ion transport accomplished by downregulation of *Slc24a4* may result in changes to neuronal excitability and calcium-dependent pathways via decreased calcium extrusion. Intriguingly, in rat PVN, this transporter has an established role in energy balance: *Slc24a4* knockout mice show hypophagia and weight loss due to constitutive activation of the melanocortin-4-receptor (Li and Lytton, 2014), a transcript which we also detected in the SFO. Importantly, altered expression of another Slc member (the Slc12a1 (Na+/K+/2Cl−) transporter) in hypothalamo-neurohypophyseal neurons causes a mild change in Cl− homeostasis but has dramatic effects on neuronal excitability and ultimately osmoregulation (Konopacka et al., 2015). The effect on calcium levels may additionally be altered by upregulation of *Cracr2b*, a regulator of intracellular CRAC Ca++ channels (Srikanth et al., 2010). Although the changes in expression of these genes are small, they have the potential to subtly alter SFO neuron electrical behaviour: SFO neurons have high input resistance, typically 2GOhm, so alteration of resting membrane currents by a few pA causes alteration of action potential activity (Fry and Ferguson 2007). Further work to determine specific actions and localisation of the Manf, Slc24a4, and Cracr2b proteins in the SFO will improve our knowledge of their electrophysiological and developmental effects, and will inform our understanding of any roles they may play in the development of negative health outcomes secondary to early postnatal overnutrition.

Related functions of differentially expressed transcripts identified using KOBAS show a trend of changes in transcripts for proteins that are involved in oxidative stress response and protein phosphorylation. Expression of heat shock protein 105 kDa (*Hsph1*), heat shock 27 kDa protein 1 (*Hspb1*), and oxidative stress regulators including *Manf* (Oh-hashi et al., 2015), cysteine-rich with EGF-like domain protein 2 (*Creld2*) (Oh-hashi et al., 2009), and cysteine and histidine rich domain containing 1 (*Chordc1*) (Gano and Simon, 2010) was significantly altered by postnatal overnutrition. Previous studies have found increased oxidative stress following early postnatal overnutrition in plasma (Habbout et al., 2012) and liver (Conceição et al., 2013). While the small litter rats in this study may not have been obese, evidence suggests they were predisposed to adult obesity (Habbout et al., 2013; Rzehak et al., 2017). While to our knowledge no studies have specifically examined the effect of early postnatal overnutrition on oxidative stress in the brain, recent research indicates a link between adult overnutrition or obesity and neural oxidative stress (Morrison et al., 2010; White et al., 2009; Zhang et al., 2005). Of considerable interest then, is whether or not the observed changes in gene expression are associated with future development of obesity, and whether or not this altered pattern of expression represents pathology or compensatory action. Additionally, it will be important to investigate whether the observed changes are unique to the SFO, unique to areas regulating energy balance and other aspect of homeostasis, or globally observed in the brains of animals subjected to early overnutrition.

Of note, the rats in this study were sacrificed peripubertally at PD 42–47. This raises a question of whether a change in onset of puberty may have played a role in the differential gene expression observed. Smith and Spencer (2018) demonstrated that the age of male puberty onset was not affected in small litters of four pups per litter, compared to litters of 12, despite their accelerated weight gain. In contrast, the age of puberty onset was significantly delayed in litters of 20 pups. Therefore, we do not believe that different age of puberty was a factor driving the observed changes in gene expression. Whether the onset of male puberty causes changes in transcription in the SFO is unknown, and to our knowledge, such changes in SFO transcription have not been reported.

Through comparing relative expression of SFO transcripts obtained via RNA sequencing in our control group to the data from the control group of a previously published microarray data (Hindmarch et al., 2008), we show correlation between data from the two techniques and validate our present dataset in a manner similar to that by other researchers comparing RNAseq and microarray datasets (Guo et al., 2013; Malone and Oliver, 2011; Mortazavi et al., 2008). We have achieved this level of replication despite that the data were produced by different laboratories, by different personnel, with rats from different suppliers and of different ages (six weeks versus 10–12 weeks). We have presented a correlation of a restricted number of transcripts (10,910). The number of transcripts shared between the two datasets was low relative to the total number of present unique transcripts found by each technique (15,937 by the microarray and 15,630 by RNAseq). This is due to limitations in mapping transcripts between the two datasets. To ensure a fair comparison, only those Affymetrix probes which could be mapped to the ENSEMBL IDs from the Rat Rnor_6.0 genome assembly were used (18,321 probes); as such, data from unannotated probes on the Affymetrix microarray could not be included (12,778 probes). This mapping issue has been encountered previously by other investigators who demonstrated similar issues with mapping Affymetrix IDs to Ensembl gene IDs (Black et al., 2014; Chen et al., 2011). A more recent microarray analysis of the rat SFO has also been published employing laser microdissection to dissect the SFO without the overlying ependymal cell layer (Szathmari et al., 2015, 2013). We expect our data to be similar to these except with regard to transcripts overrepresented within the ependymal layer, which was included in both our data and microarray data from Hindmarch et al. (2008).

The microarray study of Hindmarch et al. (2008) examining gene expression changes in rat SFO demonstrated significant changes in expression of 48 transcripts following the extreme conditions of 72 h water restriction, and 687 transcripts following 48 h food restriction. Differentially expressed transcripts included ion channels, neuropeptides and hormone receptors, and signaling molecules including neurotrophins. These results suggest the SFO is a dynamic sensor, changing its properties in response to both severe and mild challenges to homeostasis. Thus, we were intrigued when the present RNA sequencing experiment indicated that early overnutrition altered expression of only 12 transcripts, 9 of which were related to oxidative stress, and 3 transcripts with known roles in neuronal excitability and/or energy homeostasis.

Electrophysiological evidence in small litter rat neurons shows altered response to amylin in ARC and PVN (Davidowa et al., 2004) and CART in VMN (Davidowa et al., 2003; Davidowa and Plagemann, 2004), respectively. Based on this data and the sensory transduction role of SFO for circulating homeostatic signals, we anticipated changes in transcripts associated with receptors and ion channels. It is interesting then that no changes in expression of receptors and ion channels were detected. It is possible that food and water restriction used in Hindmarch et al. (2008) was a powerful and acute stimulus, whereas the early overnutrition investigated here was a mild and prolonged manipulation, not strong enough to evoke a robust changes in gene expression. Furthermore, while the reported changes are statistically significant, they are relatively subtle (<2-fold change). Thus we have come to the conclusion that early postnatal overnutrition does not cause robust changes in gene expression in the SFO such as those seen in the hypothalamus (Chen et al., 2008; Collden et al., 2015). However, some of the altered transcripts may be suited to modulate electrical excitability or neuronal differentiation. It is unclear whether these changes are reflective of early postnatal overnutrition, or whether they are predictive of changes in physiology which manifest later in life in postnatally overnourished rats, and further research along the developmental timescale will be necessary to determine this. Future research investigating the underpinnings of the effects of early postnatal overnutrition on long-term negative health outcomes should include the role of the SFO, with particular focus on the 12 transcripts we have identified.

We have for the first time examined the effect of postnatal overnutrition on the SFO transcriptome, while also producing the first RNAseq transcriptome of the SFO, building on previous microarray work (Hindmarch et al., 2008). We expect that the sequencing data presented here will provide a valuable resource for other researchers. Our raw data have been deposited in to the Sequence Read Archive (SRA) at NCBI, and we encourage other researchers to examine our data for future research investigating roles of the SFO in homeostasis.

## Declarations of interest

None.

## Disclosure

WMF and FAV designed the study. SH and SAL carried out the experiments. CSP carried out some analyses and co-wrote the first draft of the manuscript with WMF. All authors contributed to manuscript edits and approved of the final manuscript.

## Funding

This research was supported by funding from an NSERC Discovery Grant and a Research Manitoba Establishment Grant to WMF; from CIHR to AVF; from the University of Manitoba GETS program to CSP and SH; from a Research Manitoba Fellowship to SL. Funding sources had no involvement in research direction or activities.
