# Supplementary material for: The transcriptome of the rat subfornical organ is altered in response to early postnatal overnutrition
Source: IBRO Rep. 2018 Jun 26;5:17–23. doi: 10.1016/j.ibror.2018.06.001 (PMC6095096; doi:10.1016/j.ibror.2018.06.001)
Supplement: Supplementary file 1 [file mmc1.docx]

# Appendix

**Table A1.** Gene ontology analysis (generated by KOBAS) of significantly changed genes.

| Gene Ontology Term | ID | Input Number | Background Number | FDR Adjusted p-value |
| --- | --- | --- | --- | --- |
| negative regulation of oxidative stress-induced intrinsic apoptotic signaling pathway | GO:1902176 | 2 | 15 | 0.021297 |
| regulation of oxidative stress-induced intrinsic apoptotic signaling pathway | GO:1902175 | 2 | 20 | 0.021297 |
| negative regulation of oxidative stress-induced cell death | GO:1903202 | 2 | 31 | 0.021297 |
| negative regulation of response to oxidative stress | GO:1902883 | 2 | 32 | 0.021297 |
| negative regulation of cellular response to oxidative stress | GO:1900408 | 2 | 32 | 0.021297 |
| intrinsic apoptotic signaling pathway in response to oxidative stress | GO:0008631 | 2 | 33 | 0.021297 |
| chaperone-mediated protein folding | GO:0061077 | 2 | 41 | 0.024176 |
| regulation of oxidative stress-induced cell death | GO:1903201 | 2 | 41 | 0.024176 |
| regulation of cellular response to oxidative stress | GO:1900407 | 2 | 48 | 0.029084 |
| regulation of response to oxidative stress | GO:1902882 | 2 | 51 | 0.029415 |
| cell death in response to oxidative stress | GO:0036473 | 2 | 54 | 0.029854 |
| negative regulation of protein phosphorylation | GO:0001933 | 3 | 308 | 0.041021 |
| negative regulation of intrinsic apoptotic signaling pathway | GO:2001243 | 2 | 73 | 0.041997 |
| cellular response to hydrogen peroxide | GO:0070301 | 2 | 73 | 0.041997 |
| negative regulation of phosphorylation | GO:0042326 | 3 | 340 | 0.043582 |

**Figure A1**. Overlap between previously published microarray data and RNA sequencing transcriptomes. *A.* Of 15630 transcripts detected by RNAseq (compared to Rnor_6.0 rat genome assembly), 12527 transcripts could be mapped to Affymetrix IDs. 2710 could be mapped but were not expressed within the SFO according to RNAseq. *B.* Of the 15937 probes called as present on the microarray, 10910 could be mapped to 9115 unique Ensembl IDs.

5027

7411

10910

7751

Mapped to ENSEMBL ID

Present

Affymetrix Rat Gene Chip 230 (31099 probes)

12527

2710

14544

3103

Rat Rnor_6.0 (32884 gene IDs)

Mapped to Affy ID

Present

A

B

**Figure A2.** Weights of male rats from small and large (control) litters at age 6 weeks. Small litter rats were significantly heavier than controls (p < 0.05, t-test).

Large litter

(males only)

Small litter

(males only)

Weight (grams)

*
